# *Telenomuscristatus* Johnson (Hymenoptera, Scelionidae): new diagnostic data, distribution records and host associations

**DOI:** 10.3897/BDJ.11.e111347

**Published:** 2023-11-07

**Authors:** Madeline E. Potter, Jonathan S. Bremer, Matthew R. Moore, Elijah J. Talamas, Paula M. Shrewsbury

**Affiliations:** 1 University of Maryland, College Park, MD, United States of America University of Maryland College Park, MD United States of America; 2 Florida State Collection of Arthropods, Gainesville, FL, United States of America Florida State Collection of Arthropods Gainesville, FL United States of America; 3 Florida Department of Agriculture and Consumer Services, Division of Plant Industry, Molecular Diagnostics Laboratory, Gainesville, FL, United States of America Florida Department of Agriculture and Consumer Services, Division of Plant Industry, Molecular Diagnostics Laboratory Gainesville, FL United States of America

**Keywords:** egg parasitoid, BMSB, stink bug, new host associations

## Abstract

Specimens of an egg parasitoid wasp, *Telenomuscristatus* Johnson (Platygastroidea, Scelionidae), were reared from stink bug egg masses collected in the wild, in Maryland, United States. The egg masses were identified morphologically as *Halyomorphahalys* (Stål), *Banasa* Stål and *Euschistus* Dallas (Hemiptera, Pentatomidae). Molecular tools were used to further identify the *Euschistus* egg masses as *E.servus* (Say) and *E.tristigmus* (Say). All of these are new host associations for *Te.cristatus*. We also provide data to contribute to future identification of *Te.cristatus*: images of the holotype specimen and COI sequences from two disparate localities.

## New information

First record of *Te.cristatus* in Maryland and first records of it parasitizing eggs of *H.halys*, *E.servus*, *E.tristigmus* and *Banasa* sp. Images of the holotype specimen and DNA barcode data are provided.

## Introduction

*Halyomorphahalys* (Stål) (Hemiptera, Pentatomidae), also known as the brown marmorated stink bug (BMSB), is an invasive insect native to China, Japan, South Korea and Taiwan. *Halyomorphahalys* was first detected in the United States in 1996 in Allentown, Pennsylvania (Hoebeke and Carter 2003), speculated to have been brought over through bulk container shipping from Beijing, China. *Halyomorphahalys* quickly spread and is currently found in 47 U.S. states and four Canadian provinces in North America (Northeastern IPM Center 2021). This pest bug feeds on a wide range of economically important crops and woody trees with over 100 known host plants (Rice et al. 2014) and is also a nuisance for homeowners, aggregating on the outside of buildings in late autumn and overwintering inside buildings (Inkley 2012). *Halyomorphahalys* was first detected in Maryland in Washington County in 2003 (Sargent et al. 2011). In Maryland, it was initially a nuisance pest inside homes and buildings, but, as the populations grew, serious injury was reported in fruit and vegetable crops. *Halyomorphahalys* has also been reported damaging ornamental trees and shrubs, greenhouse plants and cut flowers in Maryland (Gill et al. 2010). Chemical control is currently the most widely used method for managing *H.halys*, but the broad-spectrum insecticides generally used can cause secondary pest outbreaks and compromise existing integrated pest management programs (Hull et al. 2011, Leskey et al. 2012, Rice et al. 2014).

A more sustainable approach is the use of biological control agents. Since the detection of *H.halys* in the United States, numerous studies have identified indigenous natural enemies associated with this species, the most prominent being egg parasitoids. Currently, there are 19 species of hymenopteran endoparasitoids in the genera *Anastatus* Motchulsky (Eupelmidae), *Trissolcus* Ashmead, *Telenomus* Haliday and *Hadronotus* Förster (reported as *Gryonobesum* Masner) (Scelionidae) and *Ooencyrtus* Ashmead (Encyrtidae) reported to parasitize eggs of *H.halys* in the United States (Rice et al. 2014, Abram et al. 2014, Balusu et al. 2019). To expand knowledge about the life history, geographic distribution and host associations of these parasitoids, insect egg surveys were conducted in 2020 and 2021 throughout Maryland, focused on rearing parasitoids from wild stink bug egg masses. In addition, we documented the host plant associations of the stink bug egg masses. Here, we report on new host associations of *Te.cristatus* Johnson and provide data to aid future identifications.

## Material and methods

**Insect egg surveys**: Surveys to collect naturally-laid insect eggs were conducted throughout Maryland, United States, in 2020 and 2021. In 2020, surveyors collected eggs ad hoc from commercial tree nurseries and urban woody landscapes (June through September). In 2021, fifty community scientist volunteers from the University of Maryland Extension Master Gardener Program were recruited from five Maryland counties (Allegany, Frederick, Garrett, Montgomery and Washington) and trained to help survey for eggs. Community scientists searched for and collected eggs from various habitat types (agricultural, urban herbaceous, urban vegetable garden, urban woody and woods/wooded edge) from March through September. Eggs were placed in labelled Petri dishes, which were transported in a cooler to the Shrewsbury laboratory (University of Maryland) for further processing.

**Parasitoid rearing**: Petri dishes with collected eggs were sealed with parafilm and placed into a growth chamber maintained at 23.3–25.4°C, 58–87% relative humidity (RH) and a 16L:8D photoperiod. The eggs were checked every one to six days for any emergence of stink bug nymphs or parasitoid adults from June through October 2020 and March through September 2021. Emerged parasitoids were counted and placed in labelled vials of 70% ethanol for later identification.

**Morphological identification**: All parasitoids that emerged from the eggs of Pentatomidae (stink bug) were identified to genus or species. *Telenomuspodisi* Ashmead and *Te.cristatus* were identified using the key in Johnson (1984). Pentatomidae egg masses were identified using Herbert et al. (2015), a guide by Dieckhoff (2014) and voucher specimens provided by R. A. Waterworth (USDA EPA, Washington, D.C.). Voucher specimens of *Te.cristatus* are deposited in the Florida State Collection of Arthropods, Gainesville, Florida (Table 1).

**Photography**: Images were produced with a Macropod microphography system using 10x and 20x Mitutoyo objective lenses and were rendered in Helicon focus. Images of the holotype specimen are deposited in Zenodo (https://zenodo.org/record/7709039#.ZAi_rnbMJaR). Images of molecular voucher specimens are deposited in BOLD (Barcode of Life Database), in association with their sequence and collection data.

**COI barcoding**: Genomic DNA was non-destructively isolated from entire specimens (stink bug egg masses and *Te.cristatus*) using a Qiagen DNeasy Blood and Tissue kit (Hilden, Germany). The barcode region of the mitochondrial Cytochrome c Oxidase Subunit I (CO1) was amplified using the universal barcoding primer sets LCO1490/HCO2198 (Folmer et al. 1994). PCRs used the following thermocycle: 1) initial denaturation at 95°C for 2 minutes, 2) 98°C for 30 seconds, 3) 50°C for 30 seconds, 4) 72°C for 40 seconds [32x steps 2–4] and a final extension at 72°C for 7 minutes. Egg masses from *Euschistustristigmus* required use of the primer set PENT_F2/HCO2198 (Gariepy et al. 2014). Samples were then sequenced bidirectionally on the ABI SeqStudio platform with BigDye v.3.1 chemistry. Sequences were trimmed and assembled into contigs using Geneious Prime 2023.03.

## Results

***Telenomuscristatus***

**Figure 1.**
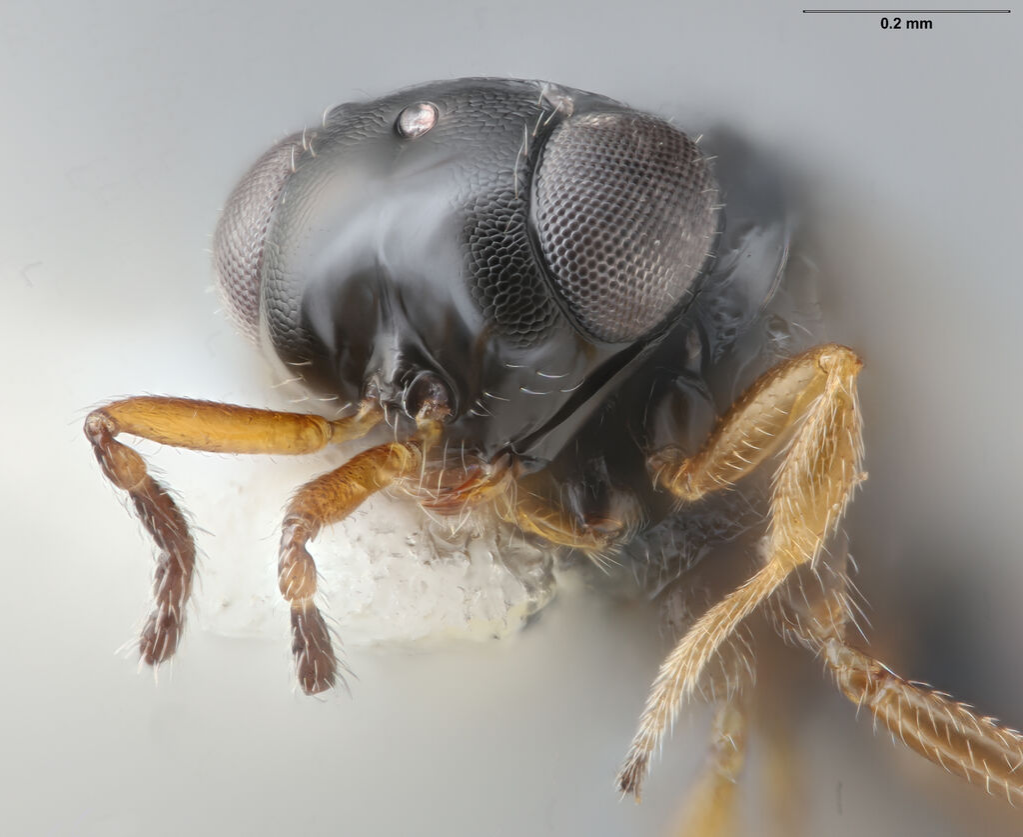
*Telenomuscristatus* (FSCA 00060141), reared from egg of *Halyomorphahalys*.

The key to species of the *Te.podisi* group in Johnson (1984) made it a straightforward task to identify the specimens that emerged from egg masses in Maryland (Figs 1, 2). These specimens are from some of the northernmost localities for *Te.cristatus*, making it worthwhile to corroborate the identification via direct comparison with the holotype specimen (Fig. 3) and comparing its COI sequences to a specimen collected in Tampa (Hillsborough County), Florida, which is relatively close to the type locality (Duval County, Florida). The high sequence identity, 99.85%, provides additional evidence that these specimens are conspecific. By providing images of the specimens used for COI sequencing (Table 1) and the holotype specimen, we expanded the available data that can be used for future identifications of *Te.cristatus*.

**Hosts**

In previous studies, *Te.cristatus* was reported to parasitize the eggs of *Chinaviahilaris* (Say), *Podisusmaculiventris* (Say) (Orr et al. 1986) and *Nezaraviridula* (Linnaeus) (Johnson 1984). We here add four new host associations, from naturally-laid eggs, for *Te.cristatus*. These were identified morphologically as *H.halys* (Stål), *Banasa* Stål, and *Euschistus* Dallas (Hemiptera, Pentatomidae) (Fig. 4). We further identified the *Euschistus* egg masses as *E.servus* (Say) and *E.tristigmus* (Say) (Table 1) by comparing COI from these egg masses with sequences in BOLD. The number of males and females of *Te.cristatus* that emerged from each egg mass are provided in Table 2.

**Stink bug egg masses**

We amplified and sequenced COI from three of the four egg masses that were morphologically identified as *Euschistus*. In BOLD, two of these matched *E.tristigmus*. Images of voucher specimens in BOLD depicted the distinctive shape of the humeral spines that characterize this species (Joe Eger, personal communication). The third specimen matched a BIN that contained sequences identified as both *E.servus* and *E.variolarius*. Many of the images associated with this BIN show the mandibular plates extending past the tylus, which is is common in northern specimens of *E.servus* and not *E.variolarius* (Joe Eger, personal communication). We therefore treat this BIN as *E.servus*. Details are provided in Table 1.

## Distribution

In the United States, *Te.cristatus* has been reported from Florida and Lousiana (Johnson 1984, Orr et al. 1986,Orr and Boethel 1990). In addition to the specimens reared in Maryland, we identified specimens of *Te.cristatus* from yellow sticky card surveys from Kentucky, North Carolina, West Virginia, Virginia and New Jersey (unpublished records). To the south, the range of this species extends at least to Mexico (Tamaulipas) and Trinidad (Johnson 1984, Orr et al. 1986, Ramirez-Ahuja et al. 2019).

## Diagnosis

Among Nearctic species of the *podisi* species group, *Te.cristatus* can be identified by the following combination of characters: hyperoccipital carina present; occiput coriaceous near hyperoccipital carina, otherwise smooth; frontal depression well developed; frons slightly bulging between antennal insertions and inner orbits; ocellar setae absent; lack of longitudinal elements in the mesoscutal sculpture; mesoscutellum with submarginal foveae smaller than metascutellar (dorsellar) punctures; greatest length of basal costae on T2 less than medial length of T1 (Figs 1, 2, 3), (Johnson 1984).

## Discussion

Numerous surveys in the United States have been conducted to assess natural enemies of *H.halys*, employing sentinel egg masses, collecting wild egg masses or a combination of both (Abram et al. 2017). Our study shows that new associations remain to be discovered and that community engagement can be a useful tool for advancing biological knowledge. By the keen eyes of Master Gardeners, we were able to collect a larger number of samples, which are needed to more thoroughly characterize parasitoid-host associations. In conjunction with the technical aspects of taxonomy and molecular diagnostics, this enabled us to advance our knowledge about the biology and distribution of *Te.cristatus*.

To date, the most dominant species of *Telenomus* associated with *H.halys* has been *Te.podisi*, which is mainly associated with field/vegetable crops and orchard habitats (Abram et al. 2017). We recovered *Te.cristatus* from a tree production nursery and the difference in the plant composition may be indicative of a habitat preference. Given that *H.halys* feeds in a variety of habitats, it is essential to select sampling sites for potential natural enemies that are equally diverse. Specimens of *Te.cristatus* in Virginia, West Virgina and North Carolina were recorded from yellow sticky cards used to survey for egg parasitoids of *H.halys*. Although these yellow sticky card surveys do not provide host information, these records have greater significance now that *Te.cristatus* is known to parasitize *H.halys* eggs. Further testing is needed to determine if these records are simply incidental and if *Te.cristatus* can parasitize *H.halys* eggs at a rate that would contribute to biological control.

We also note that the use of molecular diagnostics to identify organisms is only as accurate as the assocation between the taxon name and DNA sequence(s) used as a reference. In this study, we relied on publicly available COI sequences for species-level identification of *Euschistus* egg masses. The digital morphology framework of BOLD enabled us to enlist the help of a specialist who could interpret images associated with *Euschistus* sequences that had ambiguous identifications. In turn, we have striven to provide reliable identifications for *Te.cristatus* and associated sequences with high resolution images of vouchers.

## Figures and Tables

**Figure 2a.**
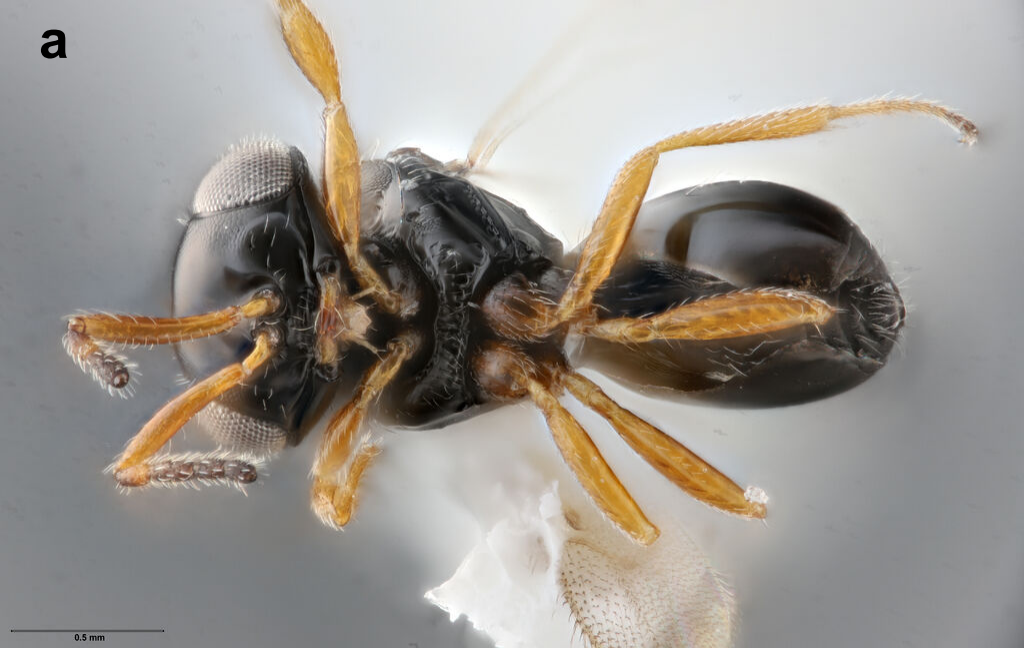


**Figure 2b.**
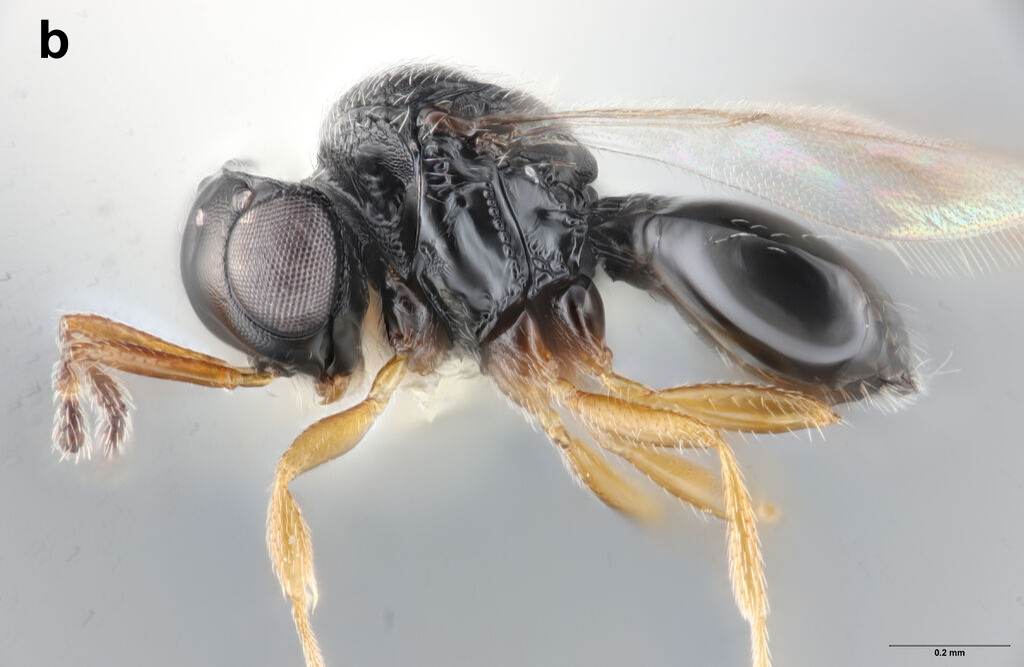


**Figure 2c.**
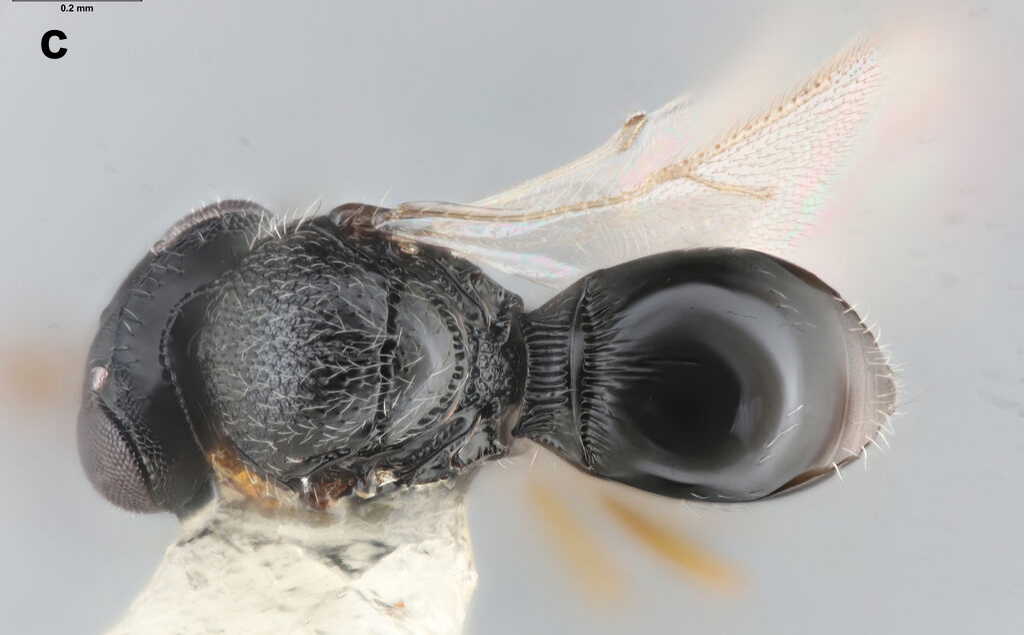


**Figure 2d.**
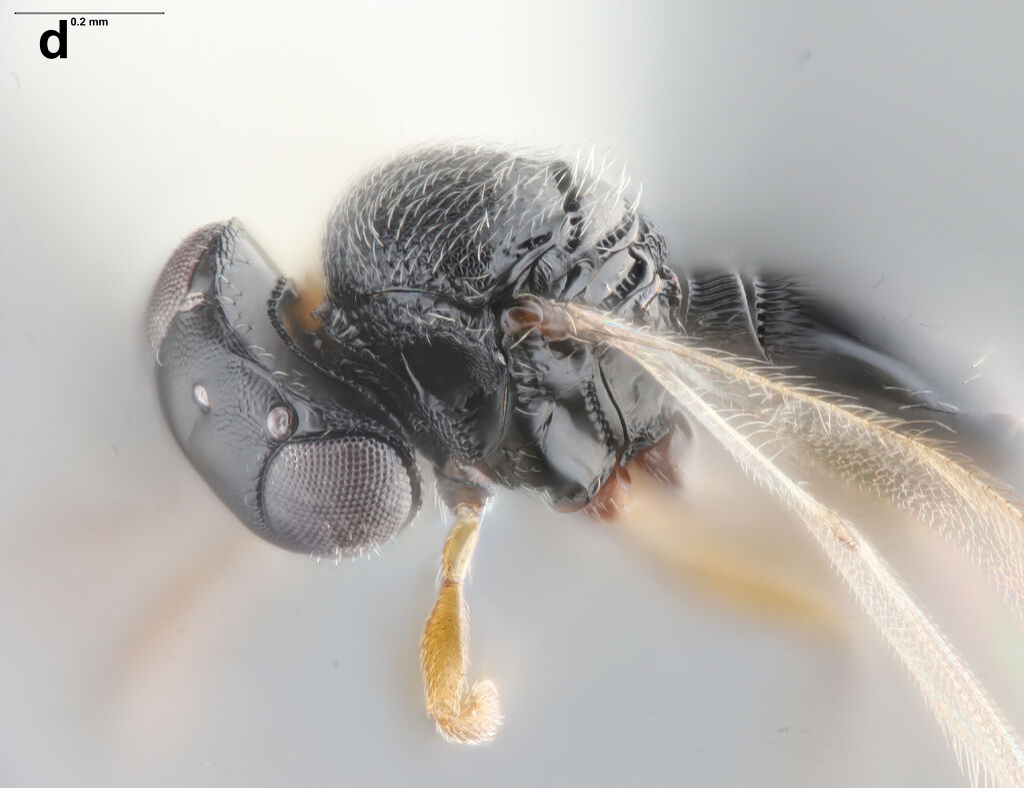


**Figure 3a.**
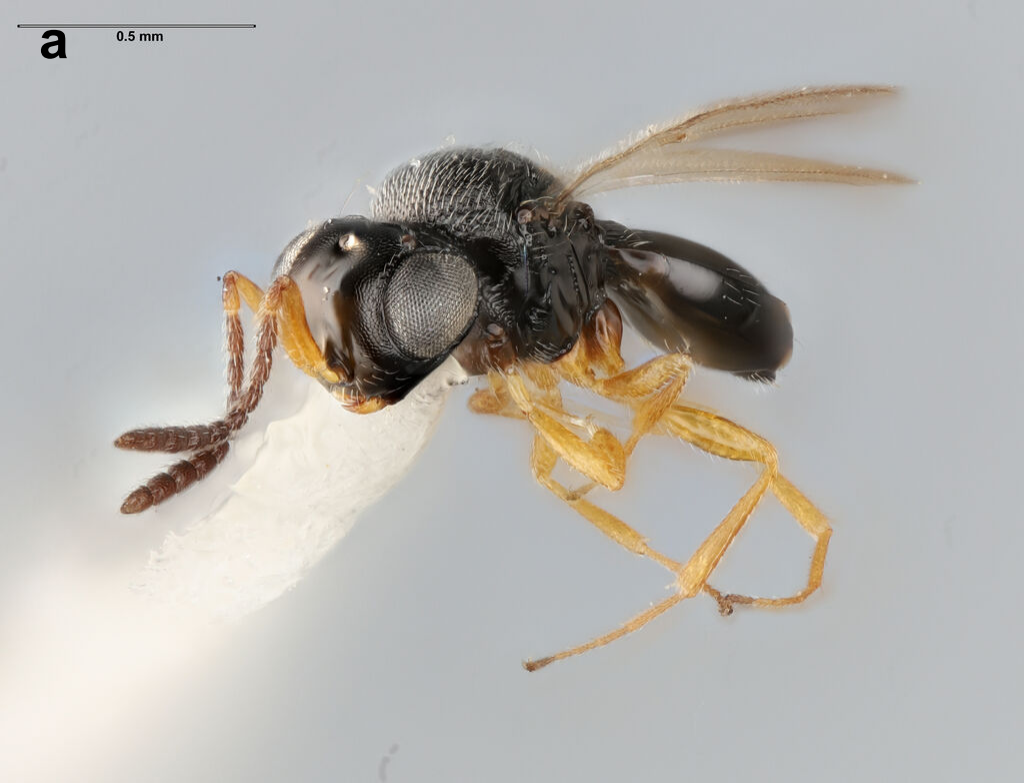


**Figure 3b.**
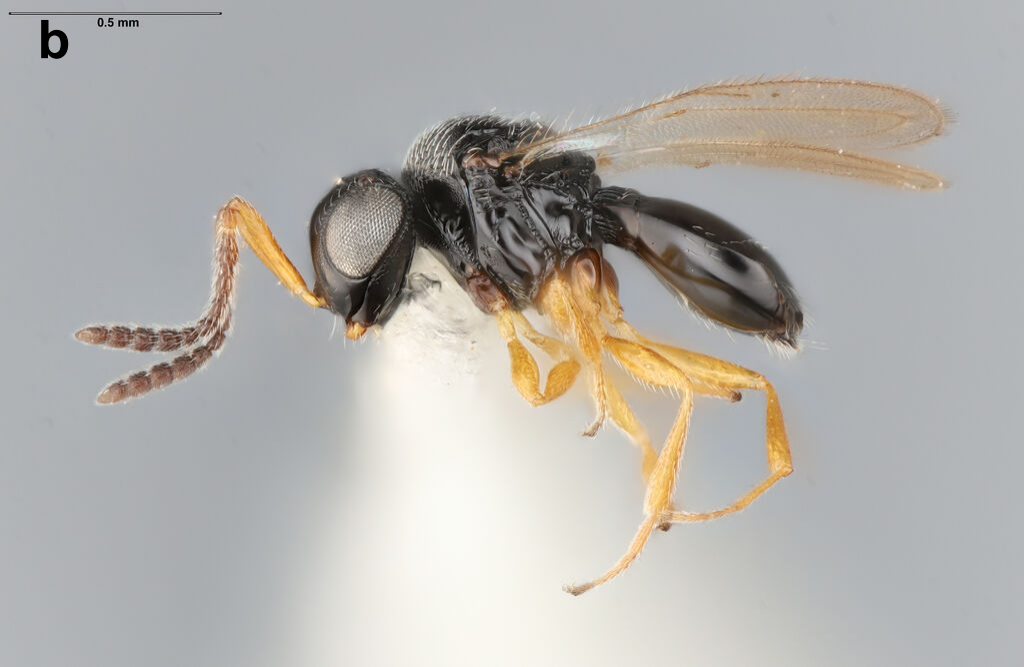


**Figure 3c.**
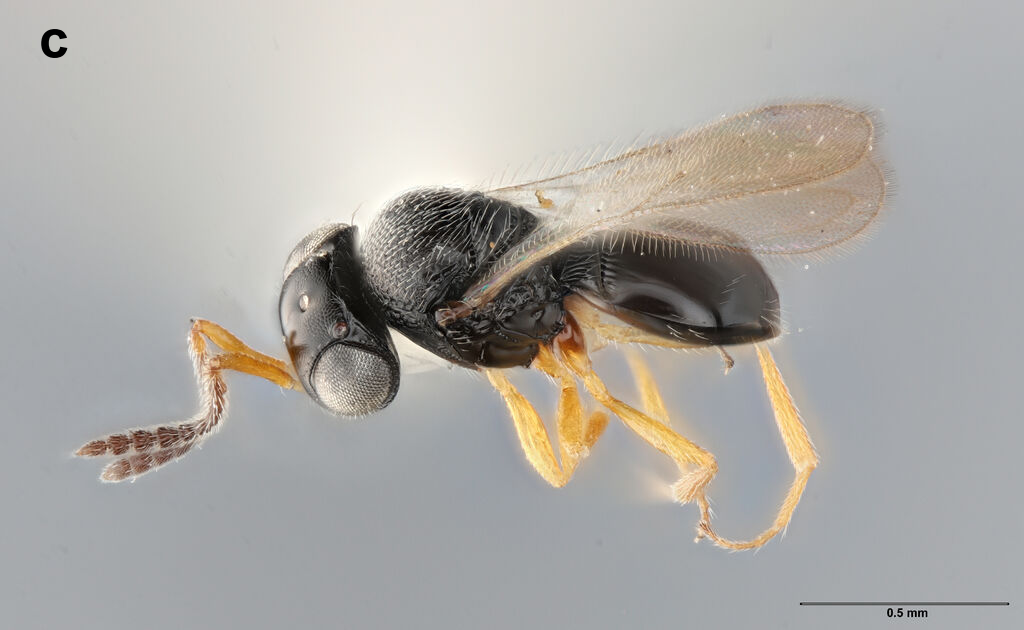


**Figure 3d.**
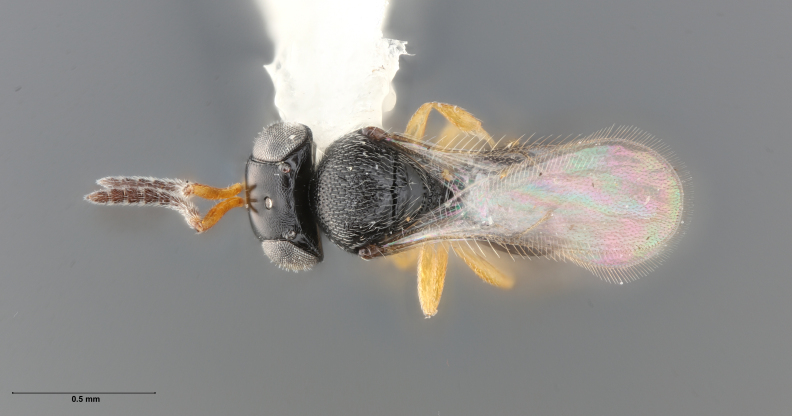


**Figure 4a.**
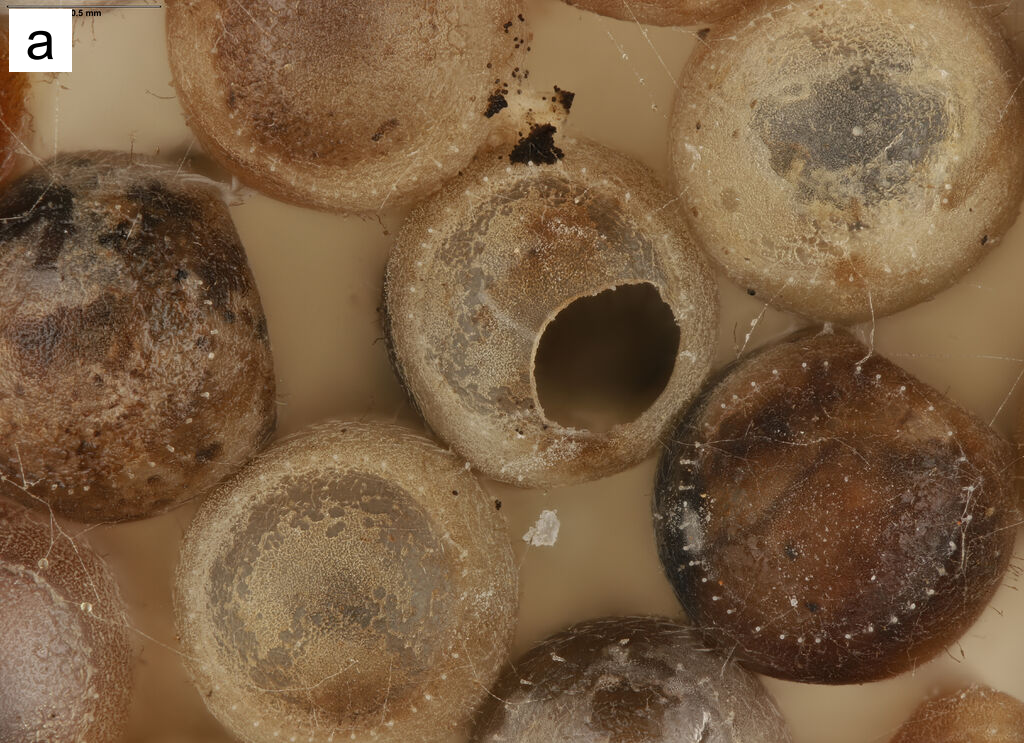
Egg mass of *Halyomorphahalys* (EM8MM);

**Figure 4b.**
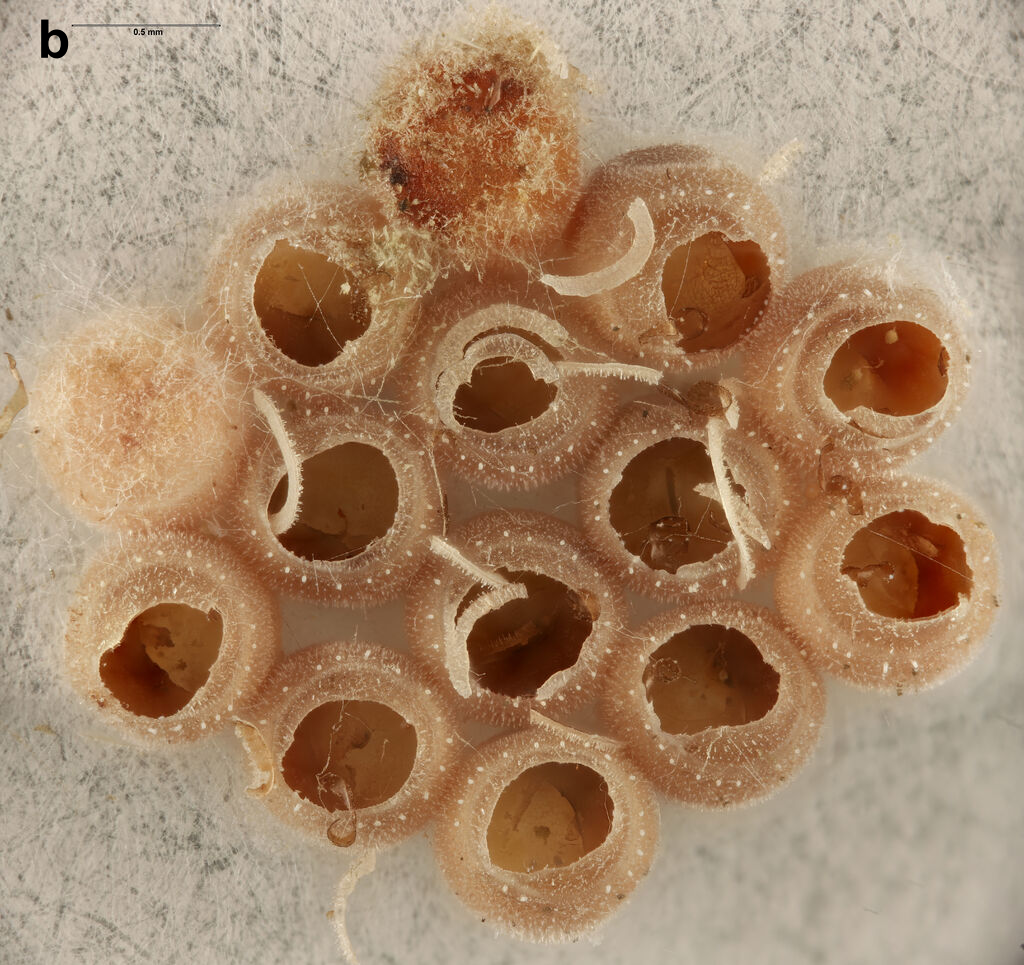
Egg mass of *Banasa* sp. (FSCA 00094204);

**Figure 4c.**
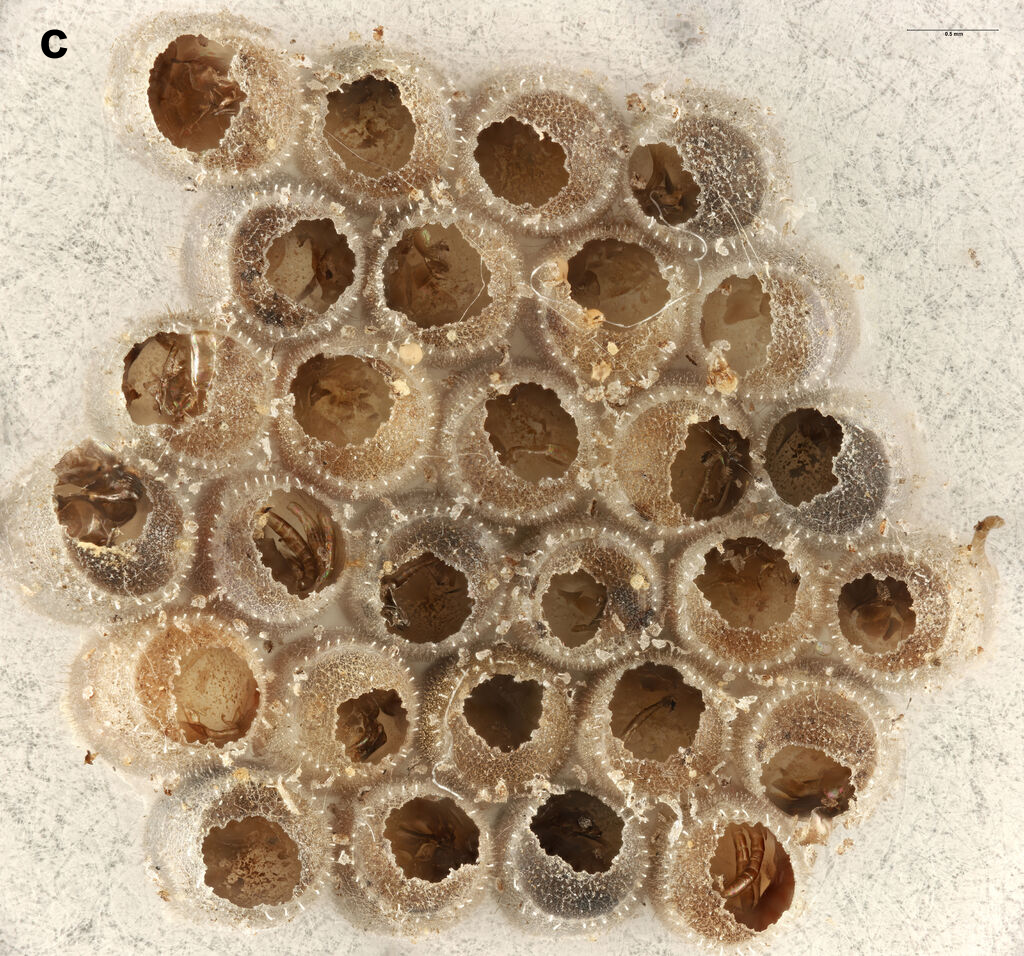
Egg mass of *E.servus* (FSCA 00094026);

**Figure 4d.**
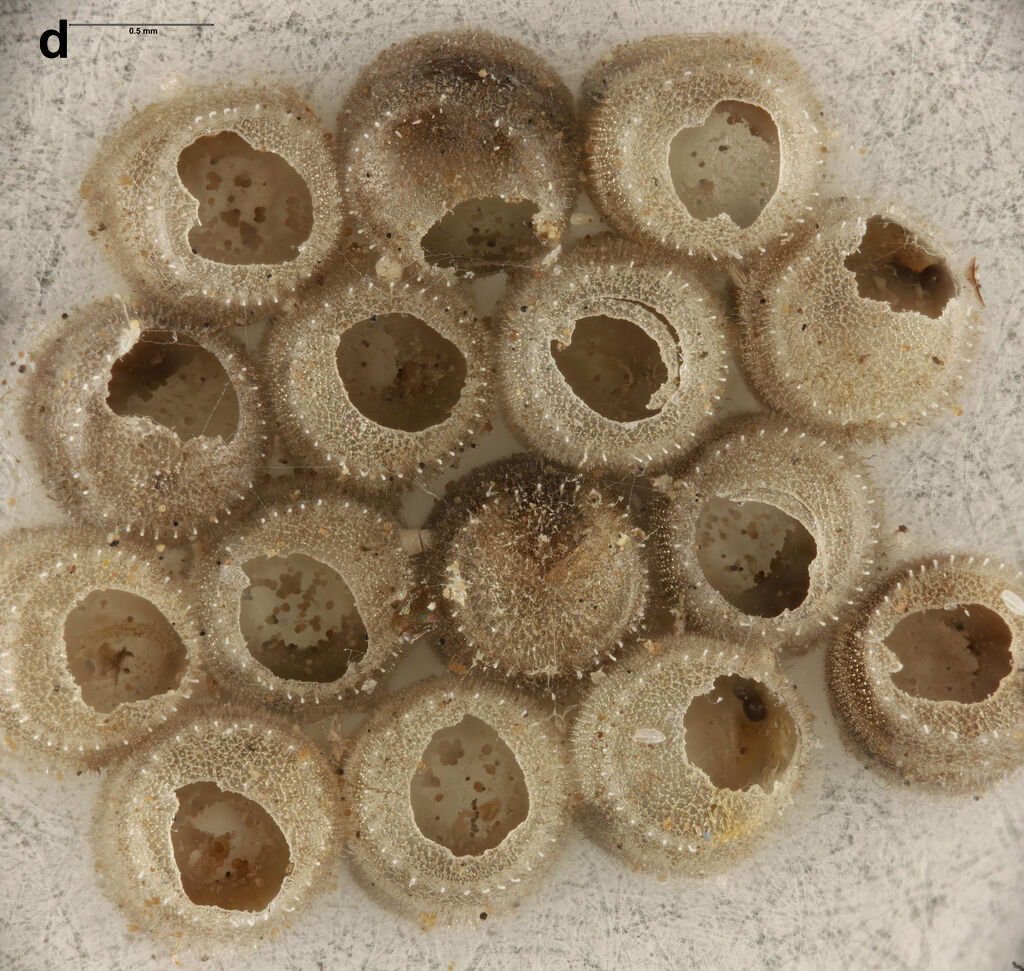
Egg mass of *E.tristigmus* (FSCA 00094028).

**Table 1.** Data associated with specimens used for COI barcoding.

**Collecting unit identifier**	**Species**	**Host**	**GenBank accession**	**BOLD BIN**
**Stink bug**				
FSCA 00094026	*Euschistusservus*	*Quercusalba*	OQ605865	BOLD:AAE0845
FSCA 00094027	*Euschistustristigmus*	*Celtisoccidentalis*	OQ605866	BOLD:AAG8876
FSCA 00094028	*Euschistustristigmus*	*Cerciscanadensis*	OQ605867	BOLD:AAG8876
**Parasitoid**				
FSCA 00060141	*Telenomuscristatus*	*Halyomorphahalys*	OP801505	
FSCA 00060144	*Telenomuscristatus*		OP801506	

**Table 2.** Emergence data for *Te.cristatus* from naturally-laid stink bug egg masses.

**Collecting unit identifier (egg mass)**	**Genus/Species**	**Total Number** **of Eggs**	***Te.cristatus*** **males**	***Te.cristatus* females**	**unsexed**
FSCA 00094024	*Banasa* sp.	14	1	11	0
FSCA 00094025	*Euschistus* sp.	20	2	3	0
FSCA 00094026	*E.servus*	28	0	1	0
FSCA 00094027	*E.tristigmus*	14	1	1	1
FSCA 00094028	*E.tristigmus*	15	1	11	0
EM8MM	*H.halys*	28	0	1	0

## References

[B8157959] Abram P. K., Gariepy T. D., Boivin G., Brodeur J. (2014). An invasive stink bug as an evolutionary trap for an indigenous egg parasitoid. Biological Invasions.

[B10088434] Abram P. K., Hoelmer K. A., Acebes-Doria A., Andrews H., Beers E. H., Bergh C. J., Bessin R., Biddinger D., Botch P., Buffington M. L., Cornelius M. L., Costi E., Delfosse E. S., Dieckhoff C., Dobson R., Donais Z., Grieshop M., Hamilton G., Haye T., Hedstrom C., Herlihy M. V., Hoddle M. S., Hooks C. R.R., Jentsch P., Joshi N. K., Kuhar T. P., Lara J., Lee J. C., Legrand A., Leskey T. C., Lowenstein D., Maistrello L., Mathews C. R., Milnes J. M., Morrison W. R., Nielson A. L., Ogburn E. C., Pickett C. H., Poley K., Pote J., Radl J., Shrewsbury P. M., Talamas E., Tavella L., Walgenbach J. F., Waterworth R., Weber D. C., Welty C., Wiman N. G. (2017). Indigenous arthropod natural enemies of the invasive brown marmorated stink bug in North America and Europe. Journal of Pest Science.

[B8157968] Balusu R. R., Cottrell T. E., Talamas E. J., Toews M. D., Blaauw B. R., Sial A. A., Buntin D. G., Vinson E. L., Fadamiro H. Y., Tillman G. P. (2019). New record of *Trissolcussolocis* (Hymenoptera: Scelionidae) parasitizing *Halyomorphahalys* (Hemiptera: Pentatomidae) in the United States of America. Biodiversity Data Journal.

[B10407421] Dieckhoff Christine (2014). Eggs of pentatomidae found in the Eastern USA Northeastern IPM. https://www.northeastipm.org/neipm/assets/File/BMSB-Resources/BMSB-Biocontrol-Workshop-Jun-2014/Eggs-of-Pentatomidae-Found-in-the-Eastern-USA-Dieckhoff.pdf.

[B8242292] Folmer O, Black M, Hoeh W, Lutz R, Vrijenhoek R (1994). DNA primers for amplification of mitochondrial cytochrome c oxidase subunit I from diverse metazoan invertebrates.. Molecular Marine Biology and Biotechnology.

[B9382321] Gariepy TD, Haye T, Zhang J (2014). A molecular diagnostic tool for the preliminary assessment of host-parasitoid associations in biological control programmes for a new invasive pest. Molecular Ecology.

[B8157992] Gill S., Klick S., Kenney S. (2010). Brown marmorated stink bug (*Halyomorphahalys*): IPM pest alert, October 2010.

[B8158002] Herbert D. A., Kamminga K., Malone S., Kuhar T. P., Day E., Greene J., Bundy C. S., Brown L., Ellsworth P. (2015). Field guide to stink bugs of agricultural importance in the United States.

[B8158015] Hoebeke E. R., Carter M. E. (2003). *Halyomorphahalys* (Stål) (Heteroptera: Pentatomidae): a polyphagous plant pest from Asia newly detected in North America. Proceedings of Entomological Society of Washington.

[B8158024] Hull L. A., Krawczyk G., Biddinger D. (2011). Maintaining the integrity of IPM in Pennsylvania while battling the brown marmorated stink bug.

[B8158032] Inkley D. (2012). Characteristics of home invasion by the brown marmorated stink bug (Hemiptera: Pentatomidae). Journal of Entomological Science.

[B8158041] Johnson N. F. (1984). Systematics of Nearctic *Telenomus*: classification and revisions of the podisi and phymatae species groups (Hymenoptera: Scelionidae). Ohio Biological Survey Bulletin New Series.

[B8158050] Leskey T. C., Short B. D., Butler B. R., Wirght S. E. (2012). Impact of the invasive brown marmorated stink bug, *Halyomorphahalys* (Stål), in Mid-Atlantic tree fruit orchards in the United States: case studies of commercial management. Psyche.

[B8158059] Center Northeastern IPM State-by-state. https://www.stopbmsb.org/where-is-bmsb/state-by-state/.

[B8158085] Orr D. B., Russin J. S., Boethel D. J., Jones W. A. (1986). Stink bug (Hemiptera: Pentatomidae) egg parasitism in Louisiana Soybeans. Environmental Entomology.

[B10088726] Orr D. B., Boethel D. J. (1990). Reproductive potential of *Telenomuscristatus* and *T.podisi* (Hymenoptera: Scelionidae), two egg parasitoids of pentatomids (Heteroptera). Annals of the Entomological Society of America.

[B8158094] Ramirez-Ahuja M., Lopez-Arroyo J. I., Rodriguez-Sanchez I. P., Gonzalez-Hernandez A. (2019). Hymenoptera parasitoids associated with Stink Bugs in Mexico. Society of Southwestern Entomologists.

[B8158105] Rice K. B., Bergh C. J., Bergmann E. J., Biddinger D. J., Dieckhoff C., Dively G., Fraser H., Gariepy T., Hamilton G., Haye T., Herbert A., Hoelmer K., Hooks C. R., Jones A., Krawczyk G., Kuhar T., Martinson H., Mitchell W., Nielsen A. L., Pfeiffer D. G., Raupp M. J., Rodriguez-Saona C., Shearer P., Shrewsbury P., Venugopal P. D., Whalen J., Wiman N. G., Leskey T. C., Tooker J. F. (2014). Biology, ecology, and management of brown marmorated stink bug (Hemiptera: Pentatomidae). Journal of Integrated Pest Management.

[B8158067] Sargent C., Martinson H. M., Raupp M. J. (2011). The orient express in Maryland: the brown marmorated stink bug, *Halyomorphahalys* (Stål) (Hemiptera: Pentatomidae). The Maryland Entomologist.

